# Laparoscopic surgery may decrease the risk of clinical anastomotic leakage and a nomogram to predict anastomotic leakage after anterior resection for rectal cancer

**DOI:** 10.1007/s00384-018-3199-z

**Published:** 2018-11-23

**Authors:** Hongtu Zheng, Zhenyu Wu, Yuchen Wu, Shanjing Mo, Weixing Dai, Fangqi Liu, Ye Xu, Sanjun Cai

**Affiliations:** 1 Department of Colorectal Surgery, Fudan University Shanghai Cancer Center, No. 270, Dong An Road, Shanghai, 200032 China; 2 Department of Oncology, Shanghai Medical College, Fudan University, Shanghai, 200032 China; 3 Department of Biostatistics, School of Public Health, Key Laboratory of Public Health Safety and Collaborative Innovation Center of Social Risks Governance in Health, Fudan University, Shanghai, China

**Keywords:** Anastomotic leakage, Rectal cancer, Risk factor, Nomogram, Laparoscopic surgery

## Abstract

**Introduction:**

Anastomotic leakage is still one of the most dreaded complications after anterior resection for rectal cancer. This study aimed to identify risk factors for anastomotic leakage and to create a nomogram for precise prediction of anastomotic leakage after anterior resection for rectal cancer.

**Methods:**

Two thousand six hundred eighteen consecutive patients who underwent anterior resection for rectal cancer with primary anastomosis, with or without diverting stoma, were retrospectively analyzed as a training dataset. Univariate and multivariable Cox regression analyses were used to determine independent risk factors associated with anastomotic leakage. A nomogram was constructed to predict anastomotic leakage. Data containing 611 patients were prospectively collected as a test dataset. The performance of the nomogram was evaluated by using a bootstrapped-concordance index and calibration plots.

**Results:**

The rate of clinical anastomotic leakage was 9.3% in the training dataset. Multivariate analysis identifies the following variables as independent risk factors for anastomotic leakage: gender (male) (odds ratio (OR) = 2.286), distance of tumor to anal verge (OR = 0.791), tumor size (OR = 1.175), operating time (OR = 1.009), diabetes mellitus (OR = 1.704), laparoscopic surgery (OR = 0.445), anastomotic bleeding (OR = 13.46), and diverting stoma (OR = 0.386). We created a nomogram with high discriminative ability (concordance index, 0.722). The area under the curve value, which evaluated the predictive performance of external validation, was 0.723.

**Conclusions:**

A protective diverting stoma and laparoscopic surgery significantly decrease the risk of anastomotic leakage. Our nomogram was a useful tool for precise prediction of anastomotic leakage after anterior resection for rectal cancer.

## Introduction

Colorectal cancer is the third most commonly diagnosed cancer and is the third leading cause of cancer-related deaths worldwide. In developing countries, the incidence of colorectal cancer has significantly increased [1]. Anterior resection (AR), as known as Dixon operation, is the major surgical treatment for rectal cancer.

Anastomotic leakage (AL) is still the most dreaded surgical complication following AR, with an incidence rate of 1.6–20.5% [2–11]. AL is associated with increased postoperative mortality, length of hospitalization, and hospital-related costs. Some of the patients with AL may require a temporary or permanent stoma, which significantly affects patients’ quality of life [12–15]. Furthermore, studies have shown that AL increases local recurrence rates and reduces cancer-specific survival [12, 16], which may be due to a delay of adjuvant therapy in patients with AL.

Many risk factors for anastomotic leakage have been reported; however, it is still difficult to predict anastomotic leakage accurately. Dekker et al. [17] retrospectively analyzed the AL risk factors in 138 patients with left-sided colon cancer and developed an AL scoring system. Frasson et al. [18] analyzed the AL risk factors in 3193 patients with colon cancer and created a devised nomogram to predict the risk of AL. However, the nomogram model can only be utilized in patients with colon cancer. Similarly, Yao et al. [9] and Kim et al. [19] constructed nomogram models as a tool for predicting the risk of AL after laparoscopic surgery of rectal cancer; however, the weights used in their reported nomogram models were different. To date, an accurate risk-predicting model for AL after AR has not been established.

The aim of this study was to create a precise and reliable nomogram for predicting the risk of AL after AR.

## Materials and methods

### Patients

Patients who underwent AR at the Shanghai Cancer Center because of rectal cancer from January 2010 to February 2016 were included in our study. Exclusion criterion included a previous history of colon or rectal resection, and patients with tumors > 12 cm from the anal verge were excluded. This study was approved by the Research Ethics Committee of the Cancer Hospital, Fudan University. Collection of patients’ follow-up data was conducted in accordance with guidelines for the collection of human follow-up data from the Cancer Hospital, Fudan University. All patients provided written informed consent.

Overall, 2618 patients were finally recruited into our following analysis to establish the nomogram model. Then, 611 patients who underwent AR from March 2016 to April 2017 in our institution were prospectively collected as a group for external validation.

### Definition of AL

Clinical anastomotic leakage was considered to be present if any of the following were observed: gas or fecal discharge from the incisional wound, vagina, or the drain tract; fecal peritonitis; or intraabdominal abscess or peritonitis along with an anastomotic defect verified by image study, sigmoidoscopy, at laparotomy, or rectal examination. A pelvic abscess near the anastomotic site without an obvious fecal fistula was also classified as a clinical leakage [4].

Based on the system proposed by the International Study Group of Rectal Cancer (ISREC), the degree of anastomotic leakage was classified into three categories: grade A required no active therapeutic intervention; grade B required active therapeutic intervention without the necessity of re-laparotomy; and grade C required re-laparotomy [20]. In the present study, clinical AL was classified as grade B or C.

### Statistical analysis

Patient-related, tumor-related, and surgery-related variables were collected as potential risk factors for AL in the univariate and multivariate analysis.

Most associations with AL, with regard to demographic and clinicopathologic features, were evaluated using multivariate logistic regression analysis. We used a stepwise selection method (sle = 0.05, sls = 0.10) to select a subset of all analyzed features. In the final model, only the features that were statistically significant at the 0.05 level were retained.

The nomogram performance was composed of two components: discrimination and calibration. The ability of a model to separate subject outcomes is known as discrimination. Discrimination was quantified with the concordance index (C-index), which is similar to the area under the receiver operating characteristic (ROC) curve [21]. Calibration was performed by comparing the predicted probability of AL versus the actual probability of AL in all patients [22], again using 500 bootstrap re-samples to reduce overfit bias, which would overstate the accuracy of the nomogram. We validated the nomograms with an external independent validation set, and the predictive performance was evaluated by the AUC value of the ROC analysis. Statistical analyses were performed using SPSS22.0, SAS 9.1, and R software version 3.1.2 (http://www.r-project.org) with the rms package. *P* values of 0.05 or lower were considered statistically significant.

## Results

A total of 2618 patients were retrospectively collected as a training dataset. The incidence of clinical AL was 9.3% (243/2618), among which the incidence rates of grade B and grade C AL were 6.3% (165/2618) and 3.0% (78/2618), respectively. Additionally, 374 patients (14.3%) received neoadjuvant radio(chemo)therapy, 395 patients (15.1%) underwent laparoscopic surgery, and 444 patients (17.0%) underwent construction of a diverting stoma (DS) during the surgery.

The following variables were found to be associated with AL in univariate analysis: distance of tumor to anal verge, tumor size, duration of operation, preoperative hemoglobin level, blood loss, male, diabetes mellitus, bowel obstruction, ASA score, laparoscopic surgery, and anastomotic bleeding. The clinical information and results of the univariate analysis of patients in the training dataset are listed in Table 1.

**Table 1:** Univariate analysis of variables related to anastomotic leakage (training dataset)

Categorical variables	All patients (*N* = 2618)	Patients without AL (*N* = 2375)	Patients with AL (*N* = 243)	*P* value
Continuous variables				
Age, year (range)	58.1 (22–93)	58.1 (22–93)	57.6 (22–81)	0.557
BMI, kg/m^2^ (range)	23.1 (14.6–38.3)	23.1 (14.6–36.0)	23.4 (15.8–38.3)	0.055
Distance of tumor to anal verge, cm (range)	11.2 (3–12)	8.1 (3–12)	7.2 (3–12)	< 0.001
Tumor size, cm (range)	3.9 (0.5–14)	3.9 (0.5–11)	4.1 (0.8–14)	0.029
Duration of operation, min (range)	117.1 (23–481)	116.1 (31–481)	128.4 (23–292)	< 0.001
Preoperative hemoglobin, g/L	130.4 (52–183)	130.1 (52–183)	133.2 (53–176)	0.010
Preoperative albumin, g/L	42.4 (26–54.4)	42.4 (26–54.4)	42.5 (29.4–54.3)	0.493
Blood lost, mL (range)	63.6 (0–1000)	64.8 (0–1000)	73.1 (0–1000)	0.034
Perioperative blood transfusion, mL (range)	6.1 (0–1000)	6.0 (0–1000)	7.0 (0–500)	0.802
Categorical variables of basic information				
Gender				< 0.001
Female	1082	1024	58	
Male	1536	1351	185	
Hypertension				0.201
No	2076	1891	185	
Yes	542	484	58	
Diabetes mellitus				0.010
No	2389	2178	211	
Yes	229	197	32	
Smoking habits				0.146
Non-smoker	2197	2001	196	
Smoker	421	374	47	
Alcohol excess				0.051
No	2384	2171	213	
Yes	234	204	30	
Bowel obstruction^a^				< 0.001
No	2585	2352	233	
Yes	33	23	10	
ASA score				0.030
1	851	759	92	
2	1192	1103	89	
3	27	24	3	
T stage				0.114
Tis	152	145	7	
T1	235	218	17	
T2	561	505	56	
T3	682	620	62	
T4a	924	833	91	
T4b	64	54	10	
N stage				0.382
N0	1496	1358	138	
N1	722	648	74	
N2	400	369	31	
M stage				0.078
M0	2413	2182	231	
M1	205	193	12	
Tumor stage (TNM-system)				0.089
Tis	151	144	7	
I	597	536	61	
II	671	608	63	
III	994	894	100	
IV	205	144	7	
Categorical variables of treatment details				
Neoadjuvant chemoradiation				0.309
No	2244	2041	203	
Yes	374	334	40	
Surgical approach				0.017
Open	2223	2004	219	
Laparoscopic	395	371	24	
Diverting stoma				0.970
No	2174	1972	202	
Yes	444	403	41	
Blood transfusion				0.572
No	2585	2346	239	
Yes	33	29	4	
Anastomotic bleeding^b^				< 0.001
No	2581	2354	227	
Yes	37	21	16	
Combined left/right hemicolectomy				0.079
No	2592	2354	238	
Yes	26	21	5	
Synchronous liver resection^c^				0.311
No	2608	2365	243	
Yes	10	10	0	

A two-tailed *P* value < 0.05 was considered statistically significant

*BMI*, body mass index; *ASA*, American Society of Anesthesiologists

^a^Bowel obstruction was defined as obvious difficulty in defecation or radiologically confirmed obstruction and dilation of the proximal bowel

^b^Anastomotic bleeding was defined as active bleeding at the anastomotic site before the end of surgery or postoperative blood stool with one or more of the following criteria: a significant fall in hemoglobin, need for blood transfusion, hemodynamic instability or shock and, finally, the need for any emergency intervention such as colonoscopy or surgery

^c^Synchronous resection of both primary tumor and liver metastasis

**Fig. 1:**
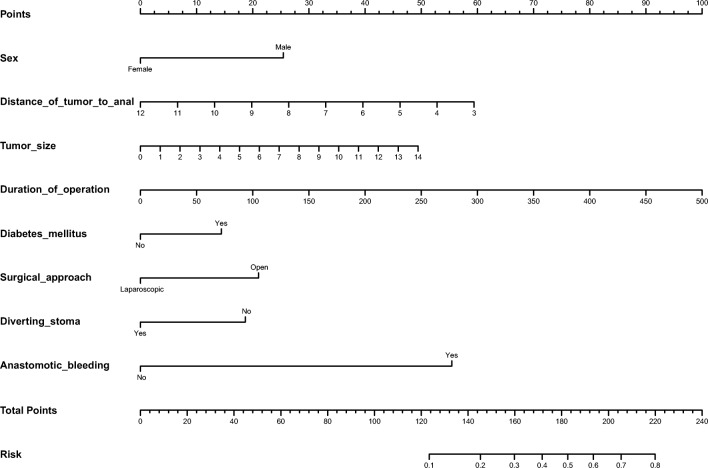
A nomogram for predicting postoperative anastomotic leakage after anterior resection for rectal cancer. To estimate the probability of AL in a given patient, mark patient values at each axis, draw a straight line perpendicular to the point axis, and sum the points for all variables. Then, we summed the total points and drew vertical line from the “total points” row to obtain the probability of anastomotic leakage

The following variables were identified as independent risk factors of AL in multivariate analysis: gender (male) (*P* < 0.0001, odds ratio (OR) = 2.286), distance of tumor to anal verge (*P* < 0.0001, OR = 0.791), tumor size (*P* = 0.006, OR = 1.175), operating time (*P* < 0.001, OR = 1.009), diabetes mellitus (*P* = 0.041, OR = 1.704), laparoscopic surgery (*P* = 0.004, OR = 0.445), anastomotic bleeding (*P* < 0.001, OR = 13.46), diverting stoma (*P* < 0.001, OR = 0.386) (Table 2). A nomogram using these risk factors as weights is shown in Fig. 1.

**Table 2:** Factors associated with anastomotic leakage after anterior resection for rectal cancer by multivariate analysis

	Adjusted OR (95% CI)	*P* value
Gender (male)	2.286 (1.484~3.520)	< 0.0001
Distance of tumor to anal verge	0.791 (0.75~0.864)	< 0.0001
Tumor size	1.175 (1.048~1.318)	0.006
Duration of operation	1.009 (1.005~1.013)	< 0.001
Diabetes mellitus	1.704 (1.023~2.837)	0.041
Surgical approach	0.445 (0.255~0.773)	0.004
Anastomotic bleeding	13.46 (5.640~31.63)	< 0.001
Diverting stoma	0.386 (0.234~0.636)	< 0.001

In the test dataset of 611 patients, the incidence of clinical AL was 10.5% (64/611). The incidence rates of grade B and grade C AL were 9.2% (56/611) and 1.3% (8/611), respectively. Additionally, 21.8% of these patients received neoadjuvant therapy (133/611), which was higher than 14.3% in the training dataset. In all, 43.4% (265/611) of these patients underwent laparoscopic surgery, which was higher than 15.1% in the training dataset. The incidence of DS in the test dataset was also higher than that in the training dataset (36.5% vs 17.0%, respectively). The reason for the differences between the two datasets dues to a more prevalent application of neoadjuvant therapy and laparoscopic surgery in our institution during the past few years, and patients who received neoadjuvant therapy or underwent low anterior resection (LAR) were more likely to receive a DS during surgery.

**Fig. 2:**
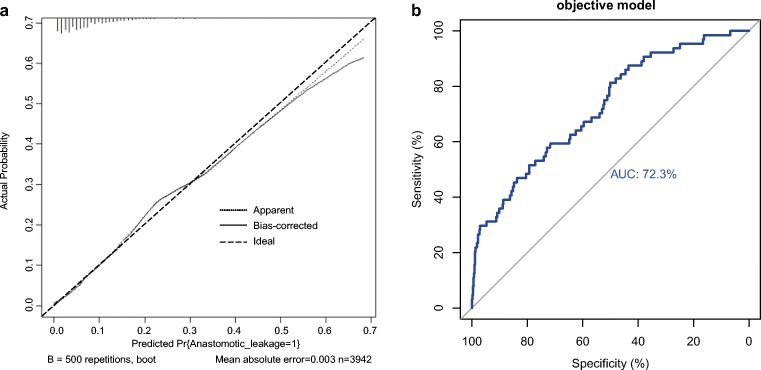
**a** A calibration plot of the predicted and observed probabilities of anastomotic leakage after anterior resection for rectal cancer. The x-axis shows the predicted probability of anastomotic leakage, and the y-axis shows the observed probability of anastomotic leakage. The nomogram had a bootstrapped-concordance index of 0.72 and was well calibrated. **b** We validated the nomograms with an external independent validation set, and the receiver operating characteristic curve for the prediction model area under the curve was 0.723

We validated the predicted efficiency of the created nomogram model using these 611 patients as a test dataset. The clinical information and identified risk factors associated with AL in the test dataset are listed in Table 3. The nomogram that integrated all objective risk factors for AL is shown in Fig. 1. The C-index for AL prediction was 0.722 (0.720–0.724) in the model. The calibration plot for the incidence of AL showed an optimal agreement between the prediction by the nomogram and the actual observation (Fig. 2). ROC analysis confirmed the diagnostic potential of the nomogram. The model yielded an AUC of 0.723.

**Table 3:** Univariate analysis of variables related to anastomotic leakage (test dataset)

Categorical variables	All patients (*N* = 611)	Patients without AL (*N* = 547)	Patients with AL (*N* = 64)	*P* value
Continuous variables				
Age, year (range)	58.7 (24–86)	58.7 (24–86)	59.1 (30–82)	0.789
BMI, kg/m^2^ (range)	23.5 (15.4–36.6)	23.5 (15.4–33.7)	23.7 (15.6–36.6)	0.572
Distance of tumor to anal verge, cm (range)	8.0 (3.5–12.0)	8.1 (3.5–12.0)	7.7 (3.5–12.0)	0.236
Tumor size, cm (range)	4.0 (0.5–12.0)	3.9 (0.5–12)	4.2 (1.5–8.0)	0.090
Duration of operation, min (range)	108.9 (27–385)	115.4 (28–385)	126.5 (50–255)	0.081
Preoperative hemoglobin, g/L	131.9 (67–170)	131.3 (67–169)	131.8 (96–170)	0.835
Preoperative albumin, g/L	43.2 (29.4–52.3)	43.3 (29.4–52.3)	42.8 (34.9–50.4)	0.353
Blood lost, mL (range)	55.0 (5–1200)	54.9 (5–1200)	56.5 (5–150)	0.841
Perioperative blood transfusion, mL (range)	6.1 (0–1000)	5.8 (0–1000)	7.8 (0–500)	0.811
Categorical variables of basic information				
Gender				< 0.001
Male	228	217	11	
Female	383	330	53	
Hypertension				0.078
No	542	481	61	
Yes	69	66	3	
Diabetes mellitus				< 0.001
No	571	521	50	
Yes	40	26	14	
Smoking habits				0.424
Non-smoker	524	467	57	
Smoker	87	80	7	
Alcohol excess				0.899
No	551	493	58	
Yes	60	54	6	
Bowel obstruction^a^				< 0.001
No	577	523	54	
Yes	34	24	10	
ASA score				0.316
1	200	173	27	
2	380	345	35	
3	24	22	2	
T stage				0.185
Tis	33	29	4	
T1	59	52	7	
T2	130	116	14	
T3	78	73	5	
T4a	283	250	33	
T4b	28	27	1	
N stage				0.372
N0	362	320	42	
N1	170	153	17	
N2	79	74	5	
M stage				0.714
M0	552	495	57	
M1	59	52	7	
Tumor stage (TNM-system)				0.676
Tis	31	27	4	
I	153	137	16	
II	149	130	19	
III	219	201	18	
IV	59	52	7	
Categorical variables of treatment details				
Neoadjuvant chemoradiation				0.536
No	478	426	52	
Yes	133	121	12	
Surgical approach				0.462
Open	346	307	39	
Laparoscopic	265	240	25	
Diverting stoma				0.860
No	388	348	40	
Yes	223	199	24	
Blood transfusion				0.741
No	604	541	63	
Yes	7	6	1	
Anastomotic bleeding^b^				< 0.001
No	598	539	59	
Yes	13	8	5	
Combined left/right hemicolectomy				0.485
No	606	543	63	
Yes	5	4	1	
Synchronous liver resection^c^				0.363
No	604	540	64	
Yes	7	7	0	

A two-tailed *P* value < 0.05 was considered statistically significant

*BMI*, body mass index; *ASA*, American Society of Anesthesiologists

^a^Bowel obstruction was defined as obvious difficulty in defecation, or radiologically confirmed obstruction and dilation of the proximal bowel

^b^Anastomotic bleeding was defined as active bleeding at the anastomotic site before the end of surgery or postoperative blood stool with one or more of the following criteria: a significant fall in hemoglobin, need for blood transfusion, hemodynamic instability or shock and, finally, the need for any emergency intervention such as colonoscopy or surgery

^c^Synchronous resection of both primary tumor and liver metastasis

## Discussion and conclusion

In this study, 2618 consecutive patients who underwent anterior resection for rectal cancer were collected to identify risk factors for anastomotic leakage. The multivariate analysis identifies the following variables as independent risk factors for anastomotic leakage: gender (male), distance of tumor to anal verge, tumor size, operating time, diabetes mellitus, laparoscopic surgery, anastomotic bleeding, and diverting stoma (Table 2). We created a nomogram with high discriminative ability.

In previous studies, there were significant variations in the incidence rates of AL after colorectal resection, ranging from 1.6 to 20.5% [2–11]. In general, the incidence of AL increases after AR compared to colon surgery. In 2016, a prospective study by Park et al. [23] showed AL rates of 5.6% (219/3912) and 1.1% (71/6565) after rectal and colon surgery, respectively. In addition, AL can be classified into asymptomatic subclinical AL and symptomatic clinical AL that requires active treatment. In 2009, ISREC classified AL into three grades (grades A, B, and C) based on its severity and required treatment regimens [20]. In 2011, Maggiori et al. [2] conducted a retrospective study that showed a total AL rate of 20.5% (41/200), of which 13.5% (27/200) accounted for clinical AL and 7% (14/200) for asymptomatic AL. In 2015, Shiomi et al. [24] reported a total AL rate of 15.9% (149/936), of which 12.9% (121/936) accounted for grades B and C. In 2016, a prospective study by Qin et al. [25] showed a total AL rate of 17.0% (54/318) after AR, with a clinical AL rate of 9.7% (31/318). However, most studies only included grades B and C. The significant difference in AL rates was attributable not only to the different ratios of the participants who underwent colon and rectal surgery but also to the inconsistent inclusion criteria for AL. In our center, the AL rates were relatively low: the incidence rate of grades B and C after resection was 6.83%, of which the incidence rates of grade B AL and grade C AL were 5.1% and 1.7%, respectively.

Our multivariate analysis showed that the postoperative risk for AL was significantly higher in male patients (adjusted OR = 2.29). Other studies obtained similar results (OR = 1.49–3.2) [5, 18, 23, 24]. The higher risk of AL in male patients may be due to their narrow pelvis, which leads to a more complicated operation compared to female patients with a broader pelvis. Some studies also showed that androgen might exert inhibitory effects on intestinal epithelial function. However, a significant association between sex and AL was not observed in other studies [25, 26]. Other patient-related risk factors, including medical history and preoperative nutritional status, were not found to be independent risk factors for AL in the present multivariate analysis.

Numerous studies found that the distance of the tumor or the anastomosis to the anal verge is closely associated with AL [5, 23, 27]. However, these studies only classified the patients into two or three groups based on the distance from the anastomosis to the anal verge. For example, Yeh et al. [4] and Jestin et al. [28] showed a significant increase in AL risk when the distance between the anastomosis and the anal verge was less than 5–6 cm. Nevertheless, these studies could not accurately predict the corresponding risk of AL based on specific tumor locations. Our study identified the corresponding risk for AL based on specific tumor locations, and a nomogram model that could accurately identify the patients at high risk for AL was then created.

In previous studies, the association between tumor size and AL was investigated. A study by Park et al. [29] showed that the AL rate was significantly higher in patients with tumor sizes > 4 cm compared with those with tumor sizes < 4 cm. In the studies conducted by Yun et al. [10] and Koyama et al. [7], the classification criteria were defined as 3 cm and 5 cm, respectively. However, no significant difference in AL risk was observed between the different groups. Nevertheless, all these studies reported higher AL rates in the patient groups with larger tumor sizes than in those with smaller tumor sizes. The absence of statistically significant differences in these studies might be due to their relatively small sample sizes. In this study, tumor size was considered a continuous variable for multivariate analysis, which showed that a larger tumor size was associated with a higher risk for AL.

The results of previous studies on whether intraoperative bleeding and blood transfusions increase the risk for AL have produced conflicting results [7, 24, 27, 28]. Our study showed that neither intraoperative bleeding nor blood transfusion increased the risk for AL. Only a few studies have investigated the relationship between AL and anastomotic bleeding, which was found in our study to be an independent risk factor for AL (adjusted OR = 11). Patients with anastomotic bleeding in both the training and validation cohorts had significantly higher risk for AL (*P* < 0.001). Anastomotic bleeding may be caused by the poor quality of the stapling device or improper use of the stapling device. All these factors may lead to increased AL risk.

The effects of neoadjuvant therapy on AL after rectal cancer resection have been controversial. In early clinical studies, most neoadjuvant radiotherapies were short-course regimens. A meta-analysis that included 7 clinical studies (including 4 with short-course radiotherapy) concluded that neoadjuvant radiotherapy did not increase the risk for AL [30]. With the increasing popularity of long-course radiotherapy as a neoadjuvant therapy, more recent clinical studies have shown that long-course neoadjuvant radiotherapy, particularly intensive chemoradiation therapy, increased the risk of AL [5, 28]. We believe that short-course radiotherapy does not cause significant bowel edema. In contrast, long-course radiotherapy, particularly intensive chemoradiation therapy, may cause significant bowel edema that increases the risk of AL. Therefore, whether the radiotherapy induced bowel edema and whether the anastomosis has been created in non-edematous areas during the surgery may be the reasons of the published discrepancy mentioned above.

A diverting stoma (DS) is often constructed to prevent AL. However, whether a DS can reduce the AL rate remains controversial. Some previous studies reported that a stoma could reduce severe anastomotic leakage and that a diverting stoma can diminish the severity of the leakage [6, 31]. In a study by Matthiessen et al. [6], 234 patients who received LAR for rectal cancer were randomly assigned to undergo DS. The AL rate of patients with a DS was 10.3%, which is significantly lower than that in patients without a DS (28%). A meta-analysis [32] which included 4 randomized controlled studies and 9 nonrandomized studies in 2015 showed that the risk of AL was significantly lower in patients with DS. However, Shiomi et al. [24] found that the presence of a stoma could not reduce the incidence rate of AL but could reduce the reoperation rate by alleviating the clinical symptoms caused by AL. Wong et al. [3] reported that a diverting stoma could not reduce grades B and C AL.

On the other hand, the impact of a DS on quality of life, such as an uncomfortable smell, the need for special care, prolapse at the stoma site, and fecal dermatitis, should not be ignored. Moreover, patients with DS are more likely to suffer from stenosis at the anastomotic site as well as significant morbidity and even mortality during stoma reversal. Several temporary stomas may become permanent [13], especially in patients who received neoadjuvant radiochemotherapy. Thus, whether a protective stoma is necessary remains controversial. We recommend identifying high-risk patients who might need colostomy using our nomogram model, which will increase the selectivity of stoma creation among patients receiving AR.

Duration of surgery is also regarded as a risk factor in some previous studies [8, 9, 17]. Our study also confirmed that patients with a longer duration of surgery had a higher risk of AL. The long duration of surgery may be caused by amateur surgical skill or poor exposure of the surgical field due to pelvic stenosis or large tumor. In addition, a decrease in blood perfusion caused by prolonged anesthesia may also increase the risk of AL.

Previous studies reported that diabetes mellitus (DM) was a risk factor for AL [11, 33]. We also got the same conclusion (OR, 1.7). The reasons why type 2 DM increased the risk of AL are as follows: insufficient blood supply to the anastomosis due to microcirculatory disorders, insufficient glycogen stores, and delayed tissue healing due to hyperglycemia. Therefore, patients with type 2 DM should be ensured that blood glucose is well controlled before surgery to reduce the risk of AL. However, several studies found that diabetes was not a risk factor for AL [27, 34].

Whether laparoscopic surgery increases the risk of anastomotic leakage is also controversial. Laparoscopic surgery does not reinforce the anastomosis conventionally, especially in low and ultralow anterior resection, which may increase the risk of anastomotic leakage. However, the CLASSIC study found that the incidence of AL was similar in laparoscopic and open surgery groups either in all enrolled patients (4% and 3%) or in the rectal cancer subgroup (8% and 7%) [35, 36]. Similarly, the COLORII study did not find that laparoscopic surgery increased the incidence of anastomotic leakage after radical resection of rectal cancer (12.6% and 10.4%, *P* = 0.462) [37]. A meta-analysis including 11 nonrandomized controlled trials and 7 randomized controlled trials reported a 7.6% incidence of anastomotic leakage after laparoscopic surgery, with no significant difference compared with 8.9% of anastomotic leakage after open surgery [38]. In our study, we found that laparoscopic surgery can reduce the incidence of anastomotic leakage. The reason may be explained by a better pelvis exposure of the surgical field, which leads to a better protection of the bowel in laparoscopic surgery.

This study had some limitations due to its retrospective nature. However, our nomogram could provide the surgeon with the precise probability of anastomotic leakage after low anterior resection for rectal cancer. Our nomogram can remind the surgeon to take precautions of patients with high AL risk. When patients with higher probability of anastomotic leakage are identified by the nomogram, they should be monitored carefully during the postoperative period. It might be helpful for them to delay removal of drainage tubes. In addition, the nomogram can also avoid an unnecessary DS in patients with low risk of AL, which reduces the quality of life and increases economic burden, as well as the risk of a permanent stoma. Our tools help to achieve more rational allocation of medical resources.

In conclusion, our study proved that a protective diverting stoma and laparoscopic surgery significantly decrease the risk of anastomotic leakage. Our nomogram was a useful tool for precise prediction of anastomotic leakage after anterior resection for rectal cancer.
